# Phenanthrene-Induced Cytochrome P450 Genes and Phenanthrene Tolerance Associated with *Arabidopsis thaliana CYP75B1* Gene

**DOI:** 10.3390/plants13121692

**Published:** 2024-06-19

**Authors:** Francisco Cabello-Hurtado, Abdelhak El Amrani

**Affiliations:** Univ Rennes, CNRS, ECOBIO, UMR 6553, Av. General Leclerc, F-35042 Rennes Cedex, France

**Keywords:** CYP75B1, 3′hydroxylated flavonoids, oxidative stress, phenanthrene, PAH tolerance

## Abstract

Polycyclic aromatic hydrocarbons (PAHs) form an important group of organic pollutants due to their distribution in the environment and their carcinogenic and/or mutagenic effects. In order to identify at the molecular level some of the players in the biodegradation and tolerance response to PAHs in plants, we have phenotyped 32 *Arabidopsis thaliana* T-DNA mutant lines corresponding to 16 cytochrome P450 (CYP) genes that showed to be differentially expressed under contrasted stress conditions induced by phenanthrene, a 3-ring PAH. This screening has allowed us to identify *CYP75B1* (At5g07990) T-DNA mutants as the only ones being sensitive to phenanthrene-induced stress, supporting that CYP75B1 protein is necessary for PAH tolerance. *CYP75B1* codes for a 3′flavonol hydroxylase. *CYP75B1* gene was heterologously expressed on yeast in order to investigate whether it affects the *A. thaliana* response to phenanthrene by participating in its metabolization. Heterologously-produced CYP75B1 enzyme shows to be catalytically efficient against its physiological substrates (e.g., naringenin) but unable to metabolize phenanthrene or 9-phenanthrenol. In contrast, CYP75B1 seems rather involved in phenanthrene tolerance as a crucial element by regulating concentration of antioxidants through the production of 3′-hydroxylated flavonoids such as quercetin and cyanidin. In particular, we report a highly increased generation of reactive oxygen species (H_2_O_2_ and singlet oxygen) in *cyp75b1* mutants compared to control plants in response to phenanthrene treatment. Overall, CYP75B1 shows to play an important role in the response to the deleterious effects of phenanthrene exposure and this is related to oxidative stress sensitivity rather than metabolization.

## 1. Introduction

Polycyclic aromatic hydrocarbons (PAHs) are widely distributed (atmosphere, water, soil and marine sediments) environmental pollutants deriving from petrogenic and pyrogenic processes of anthropogenic and/or natural origins [1]. PAHs are one of the largest known classes of chemicals with toxic, mutagenic and/or carcinogenic potential [2], sixteen of them being listed as priority pollutants by the U.S.A. Environmental Protection Agency (EPA) [3]. PAHs are, in fact, highly persistent in the environment as they are difficult to degrade and can accumulate in different compartments of the environment [4]. Although abiotic degradation and soil compartmentalization of PAHs occur, biotic metabolism is also an important route of transformation [4]. Most of the concern for public health in relation to environmental PAHs is restricted to direct exposure of humans to PAHs [5]. However, the most important biotic sink for xenobiotics in the environment is represented by plants, which amount to an estimated 85% of total living biomass [6].

PAHs have a broad impact on overall human but also plant health, both directly and by means of the reactive electrophilic metabolites obtained after PAH transformation by cells [7,8]. These reactive compounds generate oxidative stress and DNA damage, leading to cell death and/or mutations. Furthermore, because they lack an organized excretion system, plants do accumulate most of the absorbed PAHs and the metabolites formed thereof and can thus transmit these further down the food chain. Thus, available information provides evidence for the ability of plants to process and to accumulate PAHs or derived metabolites [9,10]. In contrast, much less is known concerning the underlying biochemical mechanisms, that is, the chemistry used by plants for the transformation and bioconcentration of foreign chemicals. Such knowledge is essential for developing PAH bioremediation strategies for contaminated soil and water, as well as for assessing the risk for public and environmental health linked to both the sink and metabolic capability of plants.

For several decades now, there has been general agreement that xenobiotic metabolism in plants follows a three-phase scheme [11]. Phase I consists of functionalization of a parent compound, by addition of oxygen to form a reactive site, into a less toxic product. Phase II involves conjugation of derived metabolites to a sugar, amino acid or glutathione, which increases the water solubility and reduces toxicity. Phase III involves compartmentation of derived metabolites either as soluble conjugates in the vacuole or as residues covalently bound to cell wall structures. In plants, like in animals, oxidation steps mediated by oxidative enzymes are of prime importance for PAH metabolism since they are very often rate-limiting, and they are prerequisite for further transformation [12,13].

Paramount among the xenobiotic oxidative enzymes in plants and animals are the cytochrome P450-dependent monooxygenases (CYPs), which are key enzymes in Phase I xenobiotic metabolism [11,12,13,14]. An additional aspect of CYP-mediated metabolism of organic pollutants is their participation in xenobiotic activation [14,15]. CYP-mediated xenobiotic metabolism in plants is better documented for herbicides, playing a pivotal role in their biotransformation and constituting a factor of resistance and selectivity toward herbicides [14,15,16]. Participation of plant CYPs in the metabolism of other organic chemicals has been less studied. In the case of PAHs, a few examples reporting their metabolism in microsomal fractions from several plant species are known [17,18,19,20,21,22] but participation of CYPs has only been demonstrated in some cases and, to date, no plant CYP-metabolizing PAHs have been characterized at the molecular level. However, CYP-mediated metabolism of PAHs is well described at both enzymatic and molecular level in other organisms such as humans [23], fish [24], fungi [25] and bacteria [26]. Nevertheless, plant CYPs make up about 1% of all the genes in plant genomes, constituting the largest class of plant enzymes [27]. It has thus been realized that higher plants constitute a source of CYP genes much larger in number and diversity than what is found in animals [28]. Therefore, effort focused on isolating and characterizing plant CYP genes associated with PAH metabolism is still worthy and must continue in the future. In this sense, the use of CYP effectors, sometimes successfully used to characterize and isolate plant CYPs [29,30,31], coupled to emerging omics approaches is a very promising strategy for unraveling molecular mechanisms of PAH metabolism and tolerance in plants [32].

Recently, we showed that phenanthrene tolerance is highly increased by sucrose treatment and we presented the early molecular events concomitant with tolerance to phenanthrene induced by sucrose application [33,34]. Here, we have focused on the role of the CYP complement in *A. thaliana* on sucrose-induced tolerance to phenanthrene. We have phenotyped 32 knockout mutants corresponding to 16 phenanthrene-regulated CYP genes identified after long-time [35] or short-time exposure [33,34] to phenanthrene either or not under a sucrose background. Only mutants corresponding to one of these genes (*CYP75B1*), on which we focus here, showed a modified response to phenanthrene. Further genetic, phenotypic and biochemical characterization of *CYP75B1* (At5g07990) T-DNA mutants are presented and discussed here.

## 2. Materials and Methods

### 2.1. Selection of Homozygote Lines of Arabidopsis T-DNA Insertional CYP Mutants

*Arabidopsis thaliana* T-DNA insertional mutants were obtained from the SALK, GABI-KAT and SAIL collections (Colombia (Col-0) genetic background), and from the FLAG collection (Wassilewskija (WS) background) (Table S1). Homozygous plants were selected as described by O’Malley et al. [36]. For each mutant, 16 to 20 individual plants were screened. Seed progenies of each mutant were grown on soil in the greenhouse under standard conditions, 16 h of light at 22 °C and 8 h of night at 18 °C, and two-week-old rosette leaves were harvested (3–5 mg) and used for genomic DNA extraction as described by Edwards et al. [37]. Young leaves of the same size were collected in an Eppendorf tube at room temperature without buffer for 15 s, then 400 µL of extracted buffer was added, centrifugated at 13,000 rpm for 1 min. One µL of the supernatant was used for 50 µL of standard PCR reaction. A two-steps genotyping assay was used to identify T-DNA inserts’ homozygous plants from segregating individuals. A first PCR reaction was performed to confirm that the candidate homozygous line contains a T-DNA insert at the predicted chromosomal location. Primers used were designed by SIGnAL (http://signal.salk.edu/tdnaprimers.2.html, accessed on 31 May 2021) to amplify flanking regions of the insertion site (LP + RP; Table S1). A second PCR reaction used a universal specific primer (left border LB primer) that spans the predicted T-DNA insertion site. This PCR reaction selectively amplifies the T-DNA/genomic DNA junction sequence (LB + RP).

### 2.2. Plant Growth and Chlorophyll Content

Seeds of the appropriate *Arabidopsis thaliana* line were surface sterilized and sown on Petri dishes containing half-strength Murashige and Skoog [38] medium, supplemented with sugar (either sucrose or mannitol used as osmoticum in controls) [39,40] at 3% (88 mM) for sucrose-mediated tolerance to phenanthrene studies or at 1% for mutant characterization, and either phenanthrene solution or dimethylsulfoxide (DMSO). A 700 mM phenanthrene solution was prepared in DMSO and used to provide the final required concentration in the culture medium. The same amount of DMSO was added in all treatment conditions of each experiment. Petri dishes containing seeds were placed at 4 °C over 48 h in order to break dormancy and to homogenize germination. Plants were grown at 22 °C under a 16 h light period at 110 µmol m^−2^ s^−1^. Seedlings were harvested after 8 to 22 days of growth, depending on the experiment.

Seedling fresh weight and chlorophyll content measurements were carried out as described by Shiri et al. [41]. Three biological replicates, ten plants each, were performed per treatment condition. Results represent the mean with the standard error of the mean (SEM). Statistical analyses were conducted using the Wilcoxon test by R-4.3.0 software [42].

### 2.3. Singlet Oxygen Staining

Three-week-old plantlets grown on control and treatment media were immersed and infiltrated in the dark under vacuum with a solution of 100 µM Singlet Oxygen Sensor Green^®^ reagent (SOSG) (S36002, Invitrogen, Carlsbad, CA, USA) [43] in 50 mM phosphate potassium buffer (pH 7.5). Infiltrated plantlets were then placed again on control and treatment media during 30 min in the light before being photographed under the microscope. Following excitation at 480 nm, the fluorescence emission at 530 nm was then detected by an Olympus BX41 spectrofluorometer (Shinjuku, Japan) coupled with a camera. The presence of red chlorophyll autofluorescence from chloroplasts did not alter the green fluorescence of SOSG. Experiments were repeated three times on at least 10 plantlets.

### 2.4. Hydrogen Peroxide Staining

The H_2_O_2_ staining agent, 3,3′diaminobenzidine (DAB) (D5637, Sigma-Aldrich, St. Louis, MO, USA), was dissolved in H_2_O and adjusted to pH 3.8 with KOH. The DAB solution was freshly prepared in order to avoid any auto-oxidation [44]. Three-week-old plantlets grown on control and treatment media described in legends were immersed and infiltrated under vacuum with 1.25 mg mL^−1^ DAB staining solution. Stained plantlets were then bleached in acetic acid-glycerol-ethanol (1/1/3) (*v*/*v*/*v*) solution at 100 °C for 5 min, and then stored in glycerol-ethanol (1/4) (*v*/*v*) solution until photographs were taken. H_2_O_2_ was visualized as a brown color due to DAB polymerization. Experiments were repeated three times on at least 10 plantlets.

### 2.5. CYP75B1 Yeast Expression

*Bam*HI and *Kpn*I sites (underlined in primer sequences) were introduced by PCR just upstream of the ATG and downstream of the stop codon of the full-length coding sequences (CDS), respectively, using the primers 5′-CGGGATCCATGGCAACTCTATTTCTCACAATC (sense) and 5′-GGGGTACCTTAACCCGACCCGAGTCC (reverse). The 1542-bp coding sequence (CDS) of *CYP75B1* was PCR amplified using Col-0 cDNA and TA-cloned into the pGEM-T Easy vector (Promega, Madison, WI, USA). CDS was subsequently transferred into the pYeDP60 expression vector by restriction/ligation using *Bam*HI and *Kpn*I sites. The *Saccharomyces cerevisiae* WAT11 strain was transformed with pYedP60 plasmids and selected on minimum SGI medium (20 g L^−1^ glucose, 7 g L^−1^ yeast nitrogen base, 1 g L^−1^ bactocasamino acids and 40 mg L^−1^ L-tryptophan) as described before [45]. Recombinant protein production and microsome preparation procedures were performed according to Liu et al. [45]. Briefly, 10 mL of SGI liquid culture was used to inoculate 200 mL of YPGE (10 g L^−1^ yeast extract, 10 g L^−1^ bactopeptone, 5 g L^−1^ glucose and 3% ethanol by volume) and, after 30 h growth at 28 °C, recombinant protein production was induced by addition to the medium of 10 mL of 200 g L^−1^ galactose and incubation for 16 h at 20 °C. For microsome preparation, yeast cells were harvested by centrifugation, washed with TEK buffer (50 mM Tris-HCl pH 7.5, 1 mM EDTA, 100 mM KCl) and resuspended in 2 mL of TES buffer (50 mM Tris-HCl pH 7.5, 1 mM EDTA, 600 mM sorbitol) supplemented with 5 mM 2-mercaptoethanol and 10 g L^−1^ bovine serum albumin. Cell suspensions were homogenized with 0.5 mm glass beads, cell lysates separated by 20 min centrifugation at 4 °C and 7500× *g*, and the obtained supernatant filtrated on Miracloth (22–25 µm pore size, Calbiochem, San Diego, CA, USA). Membrane microsomal fractions were then pelleted by centrifugation at 4 °C and 100,000× *g*, and resuspended in TEG buffer (50 mM Tris-HCl pH 7.5, 0.5 mM EDTA and 30% glycerol by volume) with a Potter-Elvehjem homogenizer (Thermo Fisher Scientific, Waltham, MA, USA). Microsomal membrane preparations containing CYP75B1 recombinant proteins were stored at −20 °C until processing.

### 2.6. CYP75B1 Enzyme Assays

Naringenin, phenanthrene and 9-phenantrol were obtained from Sigma-Aldrich. Naringenin, phenanthrene and 9-phenantrol metabolization was tested according to Renault et al. [46]. Briefly, standard assay composition (in 100 µL) was as follows: 50 mM KPi buffer (pH 7.4), 500 µM NADPH (omitted in control assay), 200 µM substrate, 5% DMSO (coming from substrate stock solution), 5 pmol CYP75B1 (~5 µL microsomal preparation). Assay mixtures were incubated for 30 min at 28 °C; the enzymatic reactions stopped with 10 µL 50% acetic acid and 40 µL acetonitrile. After centrifugation for 5 min at 13,000× *g*, the supernatant was recovered and analyzed by reverse-phase HPLC (Alliance 2695, Waters, Milford, MA, USA) with photo-diode array detection (DAD) (Photodiode 2996, Waters) according to Renault et al. [46]. For that purpose, 75 µL of sample was injected onto a KinetexVR core-shell C18 5 µm 4.6 × 150 mm column (Phenomenex, Torrance, CA, USA) maintained at 37 °C. The mobile phase consisted of 0.1% formic acid in water (A) and 0.1% formic acid in acetonitrile (B). Run was performed at a 1 mL min^−1^ flow rate, starting with a 5–100% B gradient (concave curve 8) for 16 min, and then isocratic conditions using 100% B for 1 min.

## 3. Results and Discussion

### 3.1. Phenotypic Characterization of Sucrose-Mediated Tolerance to Phenanthrene

**Figure 1:**
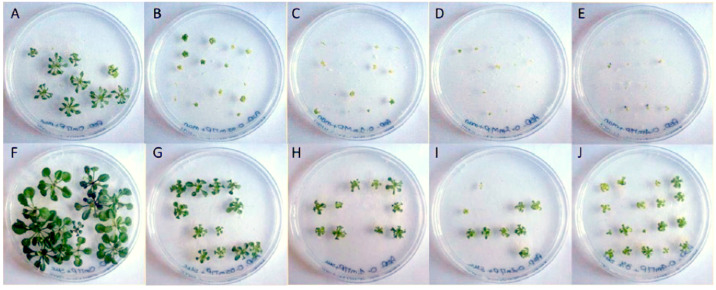
Impact of phenanthrene treatment and sucrose-mediated tolerance on *A. thaliana* rosettes. Phenotypical changes of *A. thaliana* (Col-0) rosettes under phenanthrene-induced stress with or without sucrose; 22-day-old *A. thaliana* seedlings were grown on half MS medium. Phenanthrene was supplemented at 0 μM (**A**,**F**), 50 μM (**B**,**G**), 100 μM (**C**,**H**), 200 μM (**D**,**I**) and 400 μM (**E**,**J**). Control plants (**A**–**E**) were grown on non-sucrose medium supplemented with mannitol (88 mM) as an osmoticum, while plants in pictures (**F**–**J**) were grown on 3% *w*/*v* sucrose-supplemented (88 mM) media.

We first analyzed the impact of phenanthrene on *A. thaliana* growth in the presence or absence of sucrose, and have noted that sucrose alleviates phenanthrene-induced stress. Thus, plants were exposed to different concentrations of phenanthrene (up to 400 µM) in the presence or absence of 3% (88 mM) sucrose. We found that, when phenanthrene was applied, whole seedlings and rosettes were substantially and significantly more developed in seedlings growing in a sucrose-containing medium (Figure 1, Table 1), and this from the lowest phenanthrene concentration at 50 µM. Regardless of phenanthrene concentration, the presence of sucrose in the growing medium avoided plant chlorosis and allowed leaf development (Figure 1). Thus, seedlings exposed to the highest phenanthrene concentration (400 µM) in the presence of sucrose presented a significant increase in chlorophyll content (3.0 times), which remains unchanged compared to untreated plants (153.2 ± 11.3 µg/g FW), and plant fresh weight (2.7 times) compared to seedlings grown on mannitol-supplemented media (Table 1). This capacity of sucrose to induce tolerance to phenanthrene is in good agreement with previous work showing sucrose enhanced tolerance to some organic xenobiotics [47,48].

### 3.2. Identification of Candidate CYP Genes Affecting Tolerance to Phenanthrene

The superfamily of CYP enzymes is key for xenobiotic metabolism diversity, which depends on the chemical structure of the xenobiotic compound, the organism, environmental conditions, metabolic factors and the regulated expression of these biochemical pathways [49]. Omics approaches have been used to highlight genes coding for common xenobiotic detoxification enzymes and constitute a major strategy for targeting molecular mechanisms involved in organic xenobiotic metabolism and tolerance in plants [32,50]. Previous research works have identified a range of CYP genes differentially expressed (DE) under contrasted phenanthrene-induced stress conditions (Figure S1, Table S2). Thus, we have identified a total of 41 different phenanthrene-regulated CYP genes of which 13 CYP genes were DE by a long-term phenanthrene treatment under a sucrose background (Phe+Suc vs. Such) [35], 21 after a short-term phenanthrene treatment in a sucrose-free medium (Phe vs. Control) [34], and 23 after a short-term phenanthrene treatment under a sucrose background compared to phenanthrene treatment in sucrose-free medium (Phe+Suc vs. Phe) [33].

Treatments sharing more DE CYP genes (nine genes) were Phe vs. Control and Phe+Suc vs. Phe (Figure S1). However, only one gene (*CYP89A2*) over nine presented the same behavior being inducted by both treatments (Table S2). Among the other eight shared genes, five were inhibited and three inducted in Phe+Suc vs. Phe. On the other hand, Phe vs. Control and Phe+Suc vs. Suc treatments shared five DE CYP genes, with all of them presenting the same behavior under both treatments (Table S2). All that is in good agreement with the fact that under Phe vs. Control and Phe+Suc vs. Suc situations, more stressed plants (Phe and Phe+Suc, respectively) are compared to plants in more optimal conditions (Control and Suc, respectively), whereas in Phe+Suc vs. Phe, plants in tolerance situation (Phe+Suc) are compared to stressed plants (Phe).

Eleven CYP clans are present in vascular plants, the largest ones being clan 71, by far, clan 72 and clan 85 [49]. Among the 41 DE CYPs (Table S1), 29 belong to clan 71 (12 from family CYP71, 4 from family CYP81, 3 from family CYP89, 2 from family CYP76, 2 from family CYP83 and 1 from each of the families CYP73, CYP75, CYP79, CYP98, CYP705 and CYP706), 4 to clan 72 (CYP72A subfamily), 3 to clan 85 (1 from CYP702 family and 2 from CYP708 family), 3 to clan 86 (1 from each CYP86, CYP94 and CYP704 families) and 2 to clan 710 (CYP 710A subfamily). Half of these CYP genes are of completely unknown function. Among known functions, we can find CYPs involved in the metabolism of tryptophan/camalexin, monoterpenes, phytosterols, fatty acids, gibberellins, glucosinolates and phenylpropanoids/flavonoids (Table S2).

### 3.3. Selection and Phenotyping of Arabidopsis CYP Mutants

In order to perform functional validation of phenanthrene-DE CYPs, we selected homozygous lines and phenotyped 32 T-DNA *A. thaliana* knockout mutants (Table S1) corresponding to 16 different CYPs among the 41 CYP genes responsive to short- and long-term phenanthrene treatment under either a sucrose or not a sucrose background. CYP selected genes were those for which T-DNA knockout mutants were available and whose expression was highly affected by the different phenanthrene treatments.

**Figure 2:**
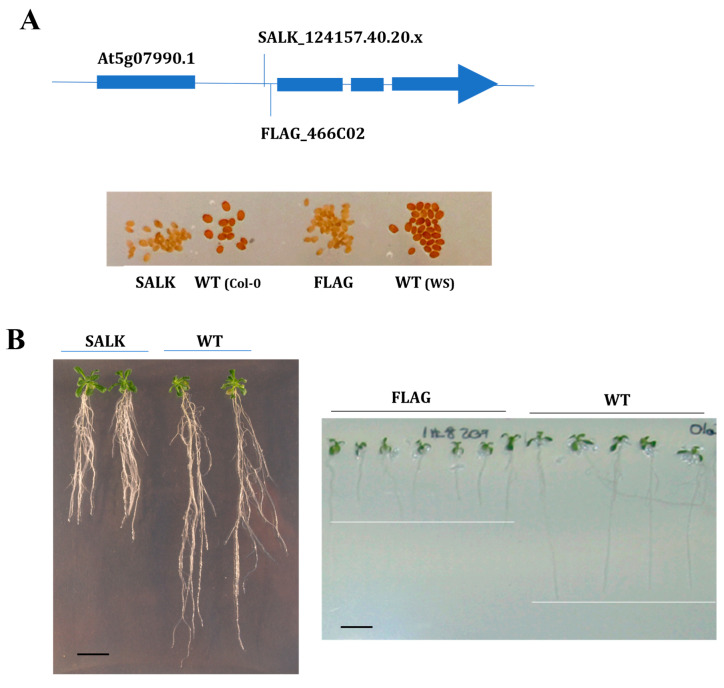
Functional validation of two alleles of At5g07990 Arabidopsis T-DNA mutants. (**A**) Homozygous T-DNA insertion mutant lines (SALK_124157.40.20.x and FLAG_466C02) inserted in the first intron of *CYP75B1* gene (At5g07990) were selected. Seeds of both mutants show transparent testa (tt) phenotype, while Col-0 (left) and WS (right) show pigmented wild-type phenotype. (**B**) Phenotypical changes of the mutants SALK_124157.40.20.x and FLAG_466C02 compared to corresponding wild-type (WT) genetic background under phenanthrene-induced (25 µM) stress. Seedlings were grown on agar medium containing 1% sucrose for 5 days, then transferred onto the indicated medium, and 21-day-old and 8-day-old representative SALK_124157.40.20.x and FLAG_466C02 plantlets were shown, respectively. Scale bars = 1 cm.

However, among all these mutants, only two of them presented increased sensitivity to phenanthrene-induced stress. Thus, only the two mutants (Figure 2A) corresponding to one of these genes, *CYP75B1* (At5g07990), showed a modified response to phenanthrene phenotype presenting a highly reduced growth under 25 µM phenanthrene (Figure 2B). Both mutant seedlings presented similar growth and morphology than the corresponding wild-type seedlings in the absence of phenanthrene stress. *CYP75B1* gene expression was only affected by a short-term phenanthrene treatment under a sucrose background compared to phenanthrene treatment in sucrose-free medium, presenting a 3.2-fold increase in expression after 8 h Phe+Suc vs. Phe (Table S2) [33]. Only two other tested CYP genes, *CYP83A1* (involved in methionine-derived aliphatic glucosinolate biosynthesis) and *CYP708A2* (triterpene thalianol hydroxylase), were also only induced under Phe+Suc vs. Phe (Table S2), but their mutants (Table S1) did not present any modified phenanthrene tolerance phenotype.

*CYP75B1* codes for a 3′flavonol hydroxylase catalyzing the conversion of naringenin to eriodictyol and of dihydrokaempferol to dihydroquercetin, and is responsible for the *A. thaliana tt7* (*transparent testa 7*) mutation [51]. In addition, it has been recently proposed that CYP75B1 is a mitochondrial CYP that, together with CYP711A1 and CYP90A1, redundantly participates in a mitochondrial ADXR–ADX–P450 (ADRX, adrenodoxin (ADR) reductase) electron transport chain that is essential for maternal gametophytic control of embryogenesis in Arabidopsis [52]. As expected, and in witnessing CYP75B1 dysfunction, seeds of both FLAG and SALK *cyp75b1* mutants presented a transparent testa phenotype (yellow/pale-brown seeds) (Figure 2A) derived from the resulting absence of dark-brown flavonoid pigments (i.e., tannins) in the seed coat [53].

### 3.4. Phenanthrene Metabolization Capacities of CYP75B1

**Figure 3:**
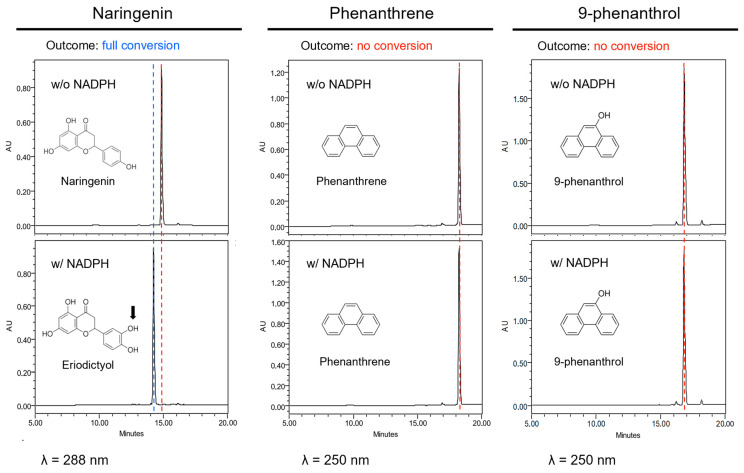
CYP75B1 metabolization assays. From left to right columns, HPLC-DAD analyses of reaction mixtures after incubation of naringenin, phenanthrene or 9-phenanthrol with a microsomal fraction from yeast cells (*Saccharomyces cerevisiae* WAT11 strain) expressing *CYP75B1* gene in the presence (lower graphics) or absence (upper graphics) of NADPH. Red dashed line, substrate; blue dashed line, product. No product was formed in any case in the absence of NADPH or with microsomes from yeast cells transformed with pYedP60 empty vector.

As natural substrates have some similarities to phenanthrene, we first hypothesized that CYP75B1 could metabolize phenanthrene into less toxic derivatives, thus explaining the increased sensitivity to phenanthrene of *cyp75b1* mutants. Therefore, microsomes prepared from yeast expressing the *CYP75B1* gene were used to assay catalytic activity on phenanthrene and 9-phenanthrol. Naringenin, a physiological substrate of CYP75B1, was tested as control. A rapid conversion into polar metabolites was only obtained with naringenin that was, as expected, converted into eriodyctiol (Figure 3). This conversion required NADPH and was absent when using yeast microsomes from yeast cells transformed with pYedP60 empty vector. Nevertheless, phenanthrene and 9-phenanthrenol were not metabolized by CYP75B1 (Figure 3). To our knowledge, at the gene level, phenanthrene-metabolizing capacities in plants have only been reported for Arabidopsis At5g05600, a putative flavone synthase belonging to the dioxygenase family [54]. In this case, Hernández-Vega et al. [54] reported a toxic accumulation of phenanthrene derivatives resulting from At5g05600 action.

### 3.5. The CYP75B1 Gene Mutant Is Affected in Oxidative Stress Response

As the functional analysis of the *CYP75B1* gene does not show metabolization of phenanthrene or 9-phenanthrenol, and phenanthrene has shown to provoke oxidative stress in plants and antioxidant mechanisms have been described as an important part of the tolerance response to phenanthrene [55,56,57], we have explored reactive oxygen species (ROS) production in *cyp75b1* mutants in response to phenanthrene.

**Figure 4:**
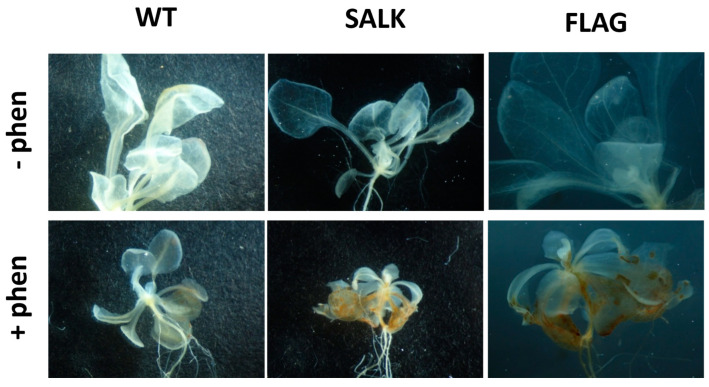
Visualization of hydrogen peroxide detected by 3,3-diaminobenzidine (DAB) staining. Detections have been carried out on 3-week-old *Arabidopsis thaliana* plantlets grown in half MS (1% sucrose) containing (+phen) or not (−phen) 50 µM phenanthrene. SALK and FLAG state for the mutant lines SALK_124157.40.20.x and FLAG_466C02, respectively, and WT for the corresponding Col-0 and WS wild-type for SALK and FLAG, respectively. Experiments were repeated three times with at least 10 plants and representative results are shown.

**Figure 5:**
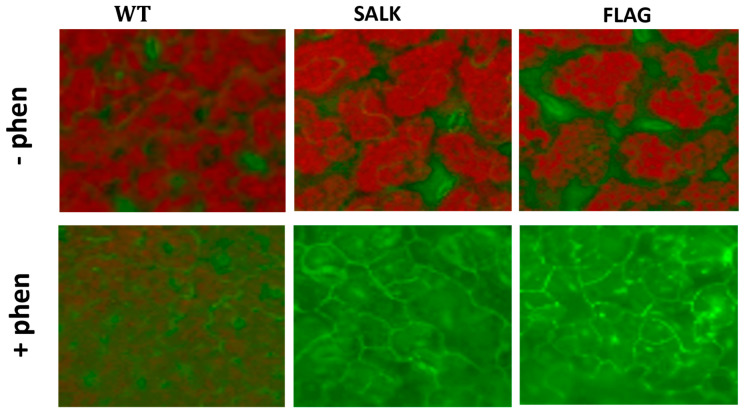
Visualization of singlet oxygen detected with the SOSG fluorescent probe. Detections have been carried out on 3-week-old *Arabidopsis thaliana* plantlets grown in half MS (1% sucrose) containing (+phen) or not (−phen) 50 µM phenanthrene. The fluorescence of SOSG corresponds to the green coloration, while the red color corresponds to chlorophyll autofluorescence. SALK and FLAG state for the mutant lines SALK_124157.40.20.x and FLAG_466C02, respectively, and WT for the corresponding Col-0 and WS wild-type for SALK and FLAG, respectively Experiments were repeated three times with at least 10 plants and representative micrographs of individual plantlets are shown.

ROS detections have been carried out on 3-week-old wild-type and *cyp75b1 A. thaliana* plantlets exposed or not to 50 µM phenanthrene. After treatment, the release of H_2_O_2_ was visualized by optical microscopy as a dark brown pigment that forms in plant tissues after reaction with DAB (Figure 4). An increase in H_2_O_2_ release in both wild-type and *cyp75b1* plants after phenanthrene treatment was observed (Figure 4). However, H_2_O_2_ levels are clearly higher in phenanthrene-stressed *cyp75b1* mutants compared to wild-type. On the other hand, singlet oxygen (^1^O_2_) in plant tissues was monitored using the SOSG fluorescent probe. Again, as observed for H_2_O_2_, even if phenanthrene treatment resulted in ^1^O_2_ production in wild-type plants, the generation of ^1^O_2_ was particularly increased in phenanthrene-stressed *cyp75b1* mutants (Figure 5). Thus, associated with reduced plant growth, phenanthrene treatment leads to an exacerbated increase in cellular generation and release of H_2_O_2_ and singlet oxygen in *cyp75b1* mutants, verifying the known increase in oxidative stress caused by phenanthrene as the result of the generation of ROS [35,57,58,59].

In view of these results, we propose that enhanced sensitivity of *cyp75b1* mutants to phenanthrene-induced stress is related to oxidative stress susceptibility. A wide spectrum of evidence points to anthocyanins as being involved in stress tolerance [60,61]. Sucrose-induced tolerance to phenanthrene could then result from sucrose up-regulation of anthocyanin biosynthetic structural genes and subsequent anthocyanin accumulation [62]. The increased ROS level in *cyp75b1* mutants could thus be explained by the absence of antioxidant 3′hydroxylated flavonoids resulting directly or indirectly from a CYP75B1 enzymatic reaction in planta. Precisely, the blockade in the 3′ hydroxylation step in *tt7* mutants, which are mutated in the *CYP75B1* gene, leads to the synthesis of pelargonidin and kaempferol instead of 3′hydroxylated cyanidin and quercetin [53,63]. In the sense of a reduced antioxidant capacity derived from altered flavonoids in *cyp75b1* mutants, it has been shown that quercetin and derivatives (dihydroxy B-ring-substituted flavonoids, i.e., presenting the catechol group in the B-ring), considered “effective antioxidants”, are by far better antioxidants than kaempferol and derivatives (monohydroxy B-ring flavonoids), which are considered “poor antioxidants”. Thus, quercetin and its derivatives are among the most powerful ROS scavengers within flavonoids, effective against stress-generated H_2_O_2_ and ^1^O_2_, for example, but also against the production of ROS by the Fenton reaction [64,65]. Furthermore, the catechol group confers on them a greater ability to modulate a stress-induced redistribution of growth [66,67].

### 3.6. Phenanthrene Sensitivity of A. thaliana Mutants Impaired in 3′-Hydroxylated Flavonoid Production

**Figure 6:**
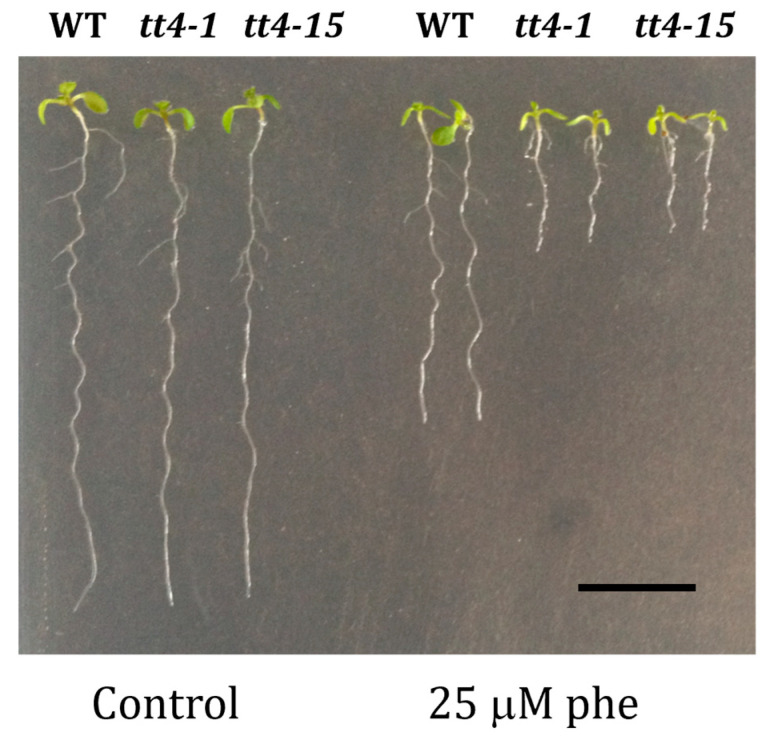
Functional validation of two alleles of the *tt4* T-DNA mutants. Homozygous T-DNA insertion mutant lines (*tt4-1* and *tt4-15*). Phenotypical changes of the mutants *tt4-1* and *tt4-15* compared to corresponding wild-type (WT) Ler genetic background, respectively, under control (0 µM phe) and phenanthrene-induced stress (25 µM phe) were performed. Seeds were grown on agar MS medium containing 1% sucrose for 5 days, then transferred onto MS medium containing or not the indicated phenanthrene concentration; 10-day-old representative *tt4-1* and *tt4-15* plantlets are shown. Scale bars = 1 cm.

In order to check whether the increased sensitivity to phenanthrene of *cyp75b1* mutants was shared by other mutants affected in 3′-hydroxylated flavonoid production, we investigated the response to phenanthrene of *tt4* mutants (two alleles, *tt4-1* and *tt4-15*), which are affected in chalcone synthase (At5g13930). These *tt4* mutants accumulate no flavonoids and are thus, together with *tt7* mutants, the only other mutants of the proanthocyanidin pathway that do not produce quercetin [53]. Consistently with this fact and the *tt7* growth phenotype in response to phenanthrene, *tt4* mutants also showed impaired growth under phenanthrene treatment (Figure 6).

In good agreement with our results, Bashandy et al. [68] described that the reduced flavonoid content conferred by *tt4* mutation provoked the reduction in tolerance to UV (UV-C) light, and Chapman and Muday [69] showed that the fluorescence level of a general ROS sensor (2′,7′-dichlorodihydro-fluorescein diacetate (CM H_2_DCF-DA) was elevated in flavonol mutants (*tt4* and *tt7*) and reduced by antioxidants (ascorbic acid). However, Chapman and Muday [69] found reduced superoxide radical (O_2_^•-^) accumulation within lateral root primordia of *tt7-2* compared with wild-type, but not in the *tt4* mutant, consistent with the opposite effects of these mutants on lateral root emergence (increased lateral root emergence in *tt4* and reduced numbers of lateral roots in *tt7-2*). In addition, antioxidant treatment reduces the lateral root number and ROS levels in *tt4*. These results support a model in which the increased level of kaempferol in the lateral root primordia of *tt7-2* reduces superoxide concentration and ROS-stimulated lateral root emergence, pointing to different antioxidant properties of 3′-hydroxylated or not 3′-hydroxylated flavonoids with different effects depending on organ and/or developmental stage.

## 4. Conclusions

Phenanthrene is a PAH molecule that negatively impacts plant growth and chlorophyll content of *A. thaliana* plants, and enhances in planta ROS production (H_2_O_2_ and ^1^O_2_). These impacts are counterbalanced by sucrose treatment, which stimulates phenanthrene tolerance through its action as a signaling molecule inducting an array of tolerance mechanisms such as antioxidant and detoxification pathways, notably including cytochrome P450s.

Here, for the first time, we have shown that CYP75B1 does not metabolize phenanthrene but its associated endogenous metabolic capacity to produce 3′-hydroxylated flavonoids (quercetin and derivatives) enhances plant growth and ROS scavenging, alleviating oxidative stress generated by phenanthrene. Accordingly, CYP75B1 and derived flavonoids could be exploited to protect plants from the oxidative damage generated by PAHs and to potentially enhance phytoremediation capacities.

## Figures and Tables

**Table 1:** Impact of phenanthrene treatment and sucrose-mediated tolerance on growth and chlorophylls of *A. thaliana* seedlings. Fresh weight (mg/seedling) and chlorophyll content (µg/g FW of seedling) of 22-day-old *A. thaliana* (Col-0) seedlings treated with 400 µM phenanthrene in the presence of 88 mM mannitol (Phe) or of 88 mM sucrose (Phe+Suc) in half MS agar medium. Values correspond to the means ± SEM of three biological replicates (ten plants each). * Indicates a significant difference between Phe- and Phe+Suc-treated plants (i.e., *p*-value < 0.05).

Fresh Weight (mg/Seedling)		Chlorophyll (µg/g FW)	
Phe	Phe+Suc	Phe	Phe+Suc
5.28 ± 0.77	14.14 ± 0.70 *	47.3 ± 8.2	142.1 ± 2.1 *

## Data Availability

Data are contained within the article and Supplementary Materials.

## Supplementary Materials

The following supporting information can be downloaded at: https://www.mdpi.com/article/10.3390/plants13121692/s1, Figure S1: Venn diagrams of differentially expressed CYPs under contrasted phenanthrene treatments. CYP genes differentially expressed under (A) a long-term (30 d) phenanthrene treatment under a sucrose background [35], (B) a short-term (up to 24 h) phenanthrene treatment in a sucrose-free medium [34] and (C) a short-term (up to 8 h) sucrose treatment under a phenanthrene background [33]; Table S1: List of Arabidopsis thaliana T-DNA insertional mutants from the SALK, GABI-KAT and SAIL collections (Col-0 genetic background) and from the FLAG collection (Wassilewskija background), and of primers used for mutant tests by PCR; Table S2: List of differentially expressed CYP genes in phenanthrene-stressed plants. Retained CYP genes were differentially expressed in short- [33] or long-term [35] protective conditions (‘phenanthrene + sucrose’ vs. ‘phenanthrene’) or phenanthrene-induced stress condition (‘phenanthrene’ vs. ‘control’) [34]. In blue, CYP genes retained for mutant analyses. log_2_ FC: Fold change (positive for up-regulation and negative for down-regulation).
