# Supplementary material for: Phenanthrene-Induced Cytochrome P450 Genes and Phenanthrene Tolerance Associated with Arabidopsis thaliana CYP75B1 Gene
Source: Plants (Basel). 2024 Jun 19;13(12):1692. doi: 10.3390/plants13121692 (PMC11207427; doi:10.3390/plants13121692)
Supplement: Supplementary file 1 [file plants-13-01692-s001.zip › Figure S1.pptx]

## Slide 1
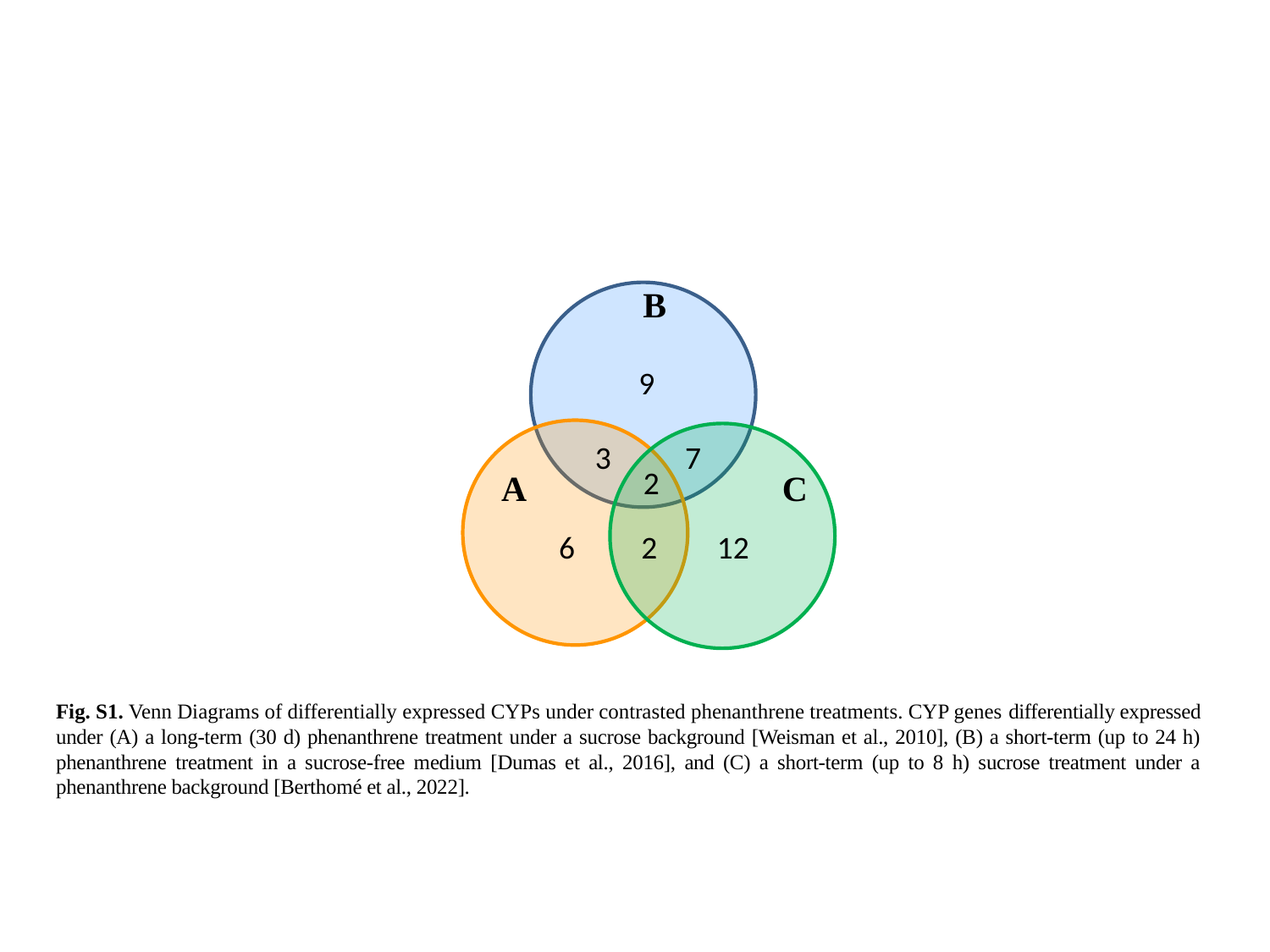

B
9
3
7
2
A
C
6
2
12
Fig. S1. Venn Diagrams of differentially expressed CYPs under contrasted phenanthrene treatments. CYP genes differentially expressed under (A) a long-term (30 d) phenanthrene treatment under a sucrose background [Weisman et al., 2010], (B) a short-term (up to 24 h) phenanthrene treatment in a sucrose-free medium [Dumas et al., 2016], and (C) a short-term (up to 8 h) sucrose treatment under a phenanthrene background [Berthomé et al., 2022].
